# Electrophysiological evidence for a neural substrate of morphological rule application in correct wordforms

**DOI:** 10.1016/j.brainres.2012.12.012

**Published:** 2013-02-16

**Authors:** Andrea Krott, Riadh Lebib

**Affiliations:** University of Birmingham, School of Psychology, Hills Building, Birmingham B15 2TT, United Kingdom

**Keywords:** Cognitive and behavioral neuroscience, Event-related potentials, Left anterior negativity, Grammatical rule application, Morphology, Inflection, German participles

## Abstract

A critical issue for understanding language processing in the brain is whether linguistic rule application is subserved by a distinct neural substrate. One of the evidence supporting this hypothesis stems from studies employing electroencephalographic measurements during the processing of rule misapplication. This evidence is inconclusive because it might reflect processes caused by the violation such as error handling rather than application of rules per se. Here we provide first evidence that correct regular formations, i.e., German past participles, are associated with left anterior negative-going activity (LAN) providing encephalographic evidence for rule application in the brain during the processing of correct words. Moreover, a LAN response is present regardless of the participles’ frequency, suggesting that independently from the mode of lexical access (i.e., decomposition or full-form activation), the cerebral structures associated with rule-based mechanisms are activated.

## Highlights

► First evidence for left anterior negativities (LANs) for correctly inflected words. ► LANs do not merely reflect the processing of grammatical errors. ► LANs are reflections of grammatical rule application per se.► LANs found for forms that could be stored as wholes and do not need rule application. ► Evidence that rule application is independent of full-form storage.

## 1 Introduction

One of the unique human capacities is the ability to produce and understand an infinite number of linguistic forms such as sentences or complex words (Corballis, 1992). Following Chomsky (1965) and Chomsky and Halle (1968), many scholars have captured this capacity by distinguishing between default rules that underlie our production and understanding of complex forms and a mental lexicon (the storage system) holding the units that the rules apply to. This has led to dual-mechanism accounts of inflectional morphology, which assume two innate but distinct neural systems: a procedural system that applies rules and a lexical storage system that stores exceptions to the rules (e.g., Clahsen, 1999; Pinker, 1999a; Pinker and Ullman, 2002; Prasada and Pinker, 1993; Ullman, 2001). For instance, the regular form *walked* is understood as a result of a mental operation that combines the stem *walk* with the regular suffix -*ed*, while the irregular form *went* is assumed to be stored in the mental lexicon. For the comprehension of inflected words, it is assumed that regular forms are decomposed into stems and affixes, while irregular forms are accessed as full-forms without decomposition.

However, this distinction between rules and lexical storage is controversial and has led to an intense debate in psychology and linguistics. Supporters of single-mechanism accounts propose that a single system handles both regular and irregular words and that rules are mere epiphenomena of similarities of forms and are not subserved by a distinct neural substrate (e.g., Joanisse and Seidenberg, 2005; McClelland and Patterson, 2002; Rumelhart and McClelland, 1986; Smolka et al., 2007). Single-systems are often modeled as connectionist networks, which represent single associative memory systems that represent both regular and irregular word forms and map, for instance, verb stems onto past tense forms (e.g., Rumelhart and McClelland, 1986).

Evidence for rule application as a separate neural process has been sought, for example, by measuring event-related potentials (ERPs) employing electroencephalography (EEG). Using a violation paradigm, i.e., presenting correctly and incorrectly formed morphological words, it has been reported that incorrect forms such as *goed* (as opposed to correct *went*), in which the *-ed* rule is incorrectly applied (rule violation), are associated with an increased left anterior negative-going activity (LAN), typically occurring between 300 and 500 ms, when compared with correct forms. However, the LAN does not occur for incorrect forms such as *bept* (as opposed to correct *beeped*), which is simply a nonword that does not violate the *-ed* rule (Gross et al., 1998; Linares et al., 2006; Morris and Holcomb, 2005; Penke et al., 1997; Rodriguez-Fornells et al., 2001; Weyerts et al., 1997). This dissociation has been found in several languages and the LAN has typically been interpreted as a cerebral response to the misapplication or violation of rules. While this dissociation suggests different processing mechanisms for regular and irregular formations, the LAN is a very indirect evidence for a distinct neural substrate for rule application because studies to date have focused on the processing of violations of rules rather than on rule application per se. The LAN might therefore merely reflect exceptional processes that are caused by the violation such as error handling and not by actual rule application. This is in line, for instance, with the suggestion that LANs might not be related to regular processes but caused by the mismatch of the presented incorrect form of a word with its stored correct form (Krott et al., 2006).

The strict distinction between decomposition for regular verbs and full-form access for irregular forms in dual-mechanism accounts has been qualified in response to studies that found an effect of the frequency of the inflected forms, i.e., of full-form frequency, on the processing of regularly inflected forms, suggesting that high-frequency regular inflections develop full-form access representations just like irregular words (e.g., Alegre and Gordon, 1999; Baayen et al., 1997; Bertram et al., 2000; New et al., 2004; Schreuder and Baayen, 1995; Sereno and Jongman, 1997). Furthermore, the recognition of low-frequency regular inflections is typically slower and leads to more errors than that of low-frequency irregular inflections, suggesting that low-frequency regular forms are recognized via the decomposition route as decomposition is believed to be slower and more error-prone than full-form activation. Full-form storage for high-frequency words is economical as it guarantees fast access of frequently occurring forms.

Previous LAN studies have not addressed the dissociation between high and low frequency regular forms observed behaviorally. If high frequency regular forms are accessed via full-form activation, rule processes might be activated only for low frequency regular forms and not for high-frequency regular forms. This would be in line with the assumption that stored forms block the application of a rule (e.g., Clahsen et al., 2004; Marcus et al., 1995; Pinker, 1999b). Alternatively, high-frequency regular forms might simultaneously activate full-form representations and are processed via decomposition, as proposed in more general dual-route models of morphological processing (Chialant and Caramazza, 1995; Niemi et al., 1994; Schreuder and Baayen, 1995). Behavioral studies cannot distinguish between these two possibilities because they cannot detect processes that do not have an impact on the behavioral response. However, evidence of such ‘silent’ process can be detected if they leave traces on the ERPs.

The present study seeks for the first time electroencephalographic evidence for rule processing by focusing on correct forms and avoiding the disadvantage of investigating the effects of rule violation. In addition, it investigates whether electrophysiological responses can confirm the behavioral dissociation between high and low frequency regular forms, by presenting both high and low frequency forms for both regular and irregular forms. Last but not least, it compared forms with correct suffixes with incorrect suffixes, in order to investigate the robustness of the LAN found for overregularisation errors (Gross et al., 1998; Linares et al., 2006; Morris and Holcomb, 2005; Penke et al., 1997; Rodriguez-Fornells et al., 2001; Weyerts et al., 1997).

We presented correct and incorrect regular and irregular German participles, which are formed by adding a prefix *ge*- to a verb stem plus either the suffix -(*e*)*t* (*ge-glaub-t* ‘believed’) or the suffix -(*e*)*n* (*ge-fahr-en* ‘driven’). Thus, we presented the correct forms *geglaubt* and *gefahren* as well as the incorrect forms *geglauben* and *gefahrt.* The prefix *ge*- is occasionally dropped when the verb contains a prefix (e.g., *unter-schrieben* ‘signed’), and some types of irregular verbs have different stem vowels for the infinitive (*schwimmen* ‘to swim’) and the past participle (*geschwommen* ‘swum’). In the present study, however, only past participles that contain the prefix *ge*- and that do not exhibit a vowel change were used. Of the two suffixes, *-*(*e*)*t* is understood as being the regular one and *-*(*e*)*n* the irregular one. This distinction is based on a number of differences between the two suffixes. Only the suffix *-(e)n* occurs with unpredictable stem vowel allomorphy (*rennen* ‘to run’—*gerannt* ‘run’ (past participle)) and therefore behaves very similar to English irregular past tense and past participles (Clahsen, 1997). During the acquisition of the participles children often overuse *-*(*e*)*t* with irregular verbs (incorrect *gekommt* instead of correct *gekommen* ‘come’), but rarely overuse *-*(*e*)*n* with regular verbs (incorrect *geschneien* instead of correct *geschneit* ‘snowed’) (Clahsen and Rothweiler, 1993). These results are closely related to the suffixes’ productivity in adult language. While *-*(*e*)*t* is used to produce participles with novel verbs (e.g., with the nonsense verb *faben*: *ge-fab-t*), *-*(*e*)*n* is very rarely used for this purpose and only if the new stem resembles an existing stem that also takes *-*(*e*)*n* (e.g., Clahsen, 1999; Marcus et al., 1995). Also, the -(*e*)*n* of low frequency participles is often replaced by *-*(*e*)*t* (e.g., Marcus et al., 1995).

We focused on German past participles for two reasons. First, unlike English irregular verbs which tend to occur in phonological clusters such as *drink, sink, sing* etc. that help to predict the form of similar past participles (*drunk, sunk, sung* for the above mentioned example), German verb stems do not provide any reliable indication of whether they are irregular or regular (Beedham, 1994). This means that only the suffix *-*(*e*)*t* can be described by means of a rule. Second and more importantly, studies on the English past tense have suggested that processing differences between regular and irregular past tense forms might be due to differences in phonological complexity (Bird et al. 2003; McClelland and Patterson, 2002). Adding the regular suffix *-ed* to English past tense forms increases the complexity of the forms, while changing the stem vowel, which is typical for irregular past tense forms (e.g., *drink—drank*), does not add complexity. In contrast, German regular and irregular participles are all concatenative and differ only in terms of the suffix (-(*e*)*t* versus -(*e*)*n*). Therefore, any differences between regular and irregular forms in our experiment cannot be due to differences in phonological complexity.

With respect to correct participle forms, we expected the following. If LANs reflect rule application, then correct regular participles (*geglaubt)* should exhibit left anterior activations that are more negative than those for correct irregular participles (*gefahren*). If high-frequency regular forms are accessed via their full-forms, as suggested in previous studies (e.g., Alegre and Gordon, 1999; Baayen et al., 1997; Bertram et al., 2000; New et al., 2004; Schreuder and Baayen, 1995; Sereno and Jongman, 1997), and if full-from access blocks rule application, then LANs should occur only for correct low frequency regular participles, not for correct high-frequency regular participles. If, on the other hand, regular forms activate rules independently of full-form access then LANs should occur for both high and low frequency regular participles.

With respect to incorrect participle forms, we expected to replicate the findings of Penke et al. (1997), who found a LAN (i.e., a left frontotemporal negativity) for incorrect (i.e., overregularised) irregular forms (*gefahrt*) compared to their correct forms (*gefahren*) and no difference between incorrect (*geglauben*) and correct (*geglaubt)* regular forms. For German noun plurals, incorrect regular forms have led to increased N400 responses (Weyerts et al., 1997). We might therefore also see an increased N400 component for incorrect participles. Most importantly, though, we expected that the incorrect use of regular versus irregular suffixes elicit distinct scalp distributions.

Apart from LANs, we investigated whether our frequency contrast between high and low frequency participles was large enough to allow us to detect processing differences with regard to regularity. We expected an N400 effect, with low frequency words evoking larger negative amplitudes than high frequency words over central-posterior sites around 400 ms (e.g., Barber et al., 2004; Dambacher et al., 2006; Rugg, 1990; Van Petten and Kutas, 1990).

## 2 Results

**Fig. 1:**
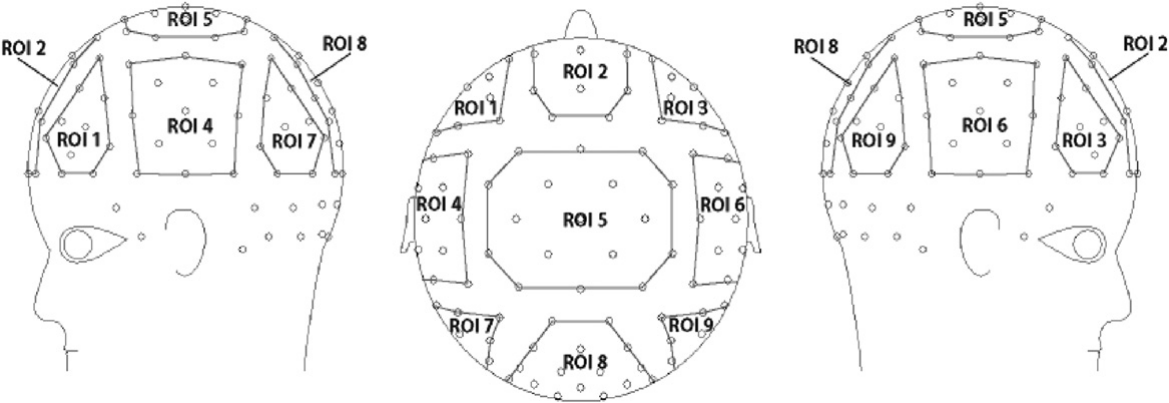
Localization of the 9 regions of interest (ROIs) over the scalp.

Participants’ error rates were well below 5%, showing that they were attending closely to the stimuli. Behavioral data will therefore not be discussed further. For the ERP analyses, we focused on two consecutive time-windows, 300–350 ms and 350–400 ms, which showed qualitative differences with respect to LAN effects. These windows were also suited to investigate the N400 component. In order to capture the scalp distribution of both LAN and N400 effects as left anterior (temporal) effects and central/posterior effects, respectively, we divided the electrodes symmetrically into nine clusters (ROIs) keeping the number of electrodes in each cluster as similar as possible. We then averaged the values of the electrodes within these clusters. Fig. 1 illustrates the topographical position of the ROIs, and Table 1 lists the electrodes we pooled for each ROI.

### 2.1 Correct regular vs. irregular participle forms

To investigate whether correct regular participles show a LAN compared to correct irregular participles, we conducted ANOVAs on ERPs to correct participles with the factors Regularity (regular vs. irregular), Frequency (high frequency vs. low frequency), and ROI (9 levels) for both time-windows. In this and the following analyses, the estimated Greenhouse–Geisser coefficient epsilon was applied when necessary to correct for violations of the assumption of sphericity, and *p* values are reported according to the corrected degrees of freedom. Finally, we used Bonferroni-corrected *α* levels when applicable (e.g., when testing an effect in each ROI). The results of the ANOVAs for both time-windows are listed in Table 2.

**Fig. 2:**
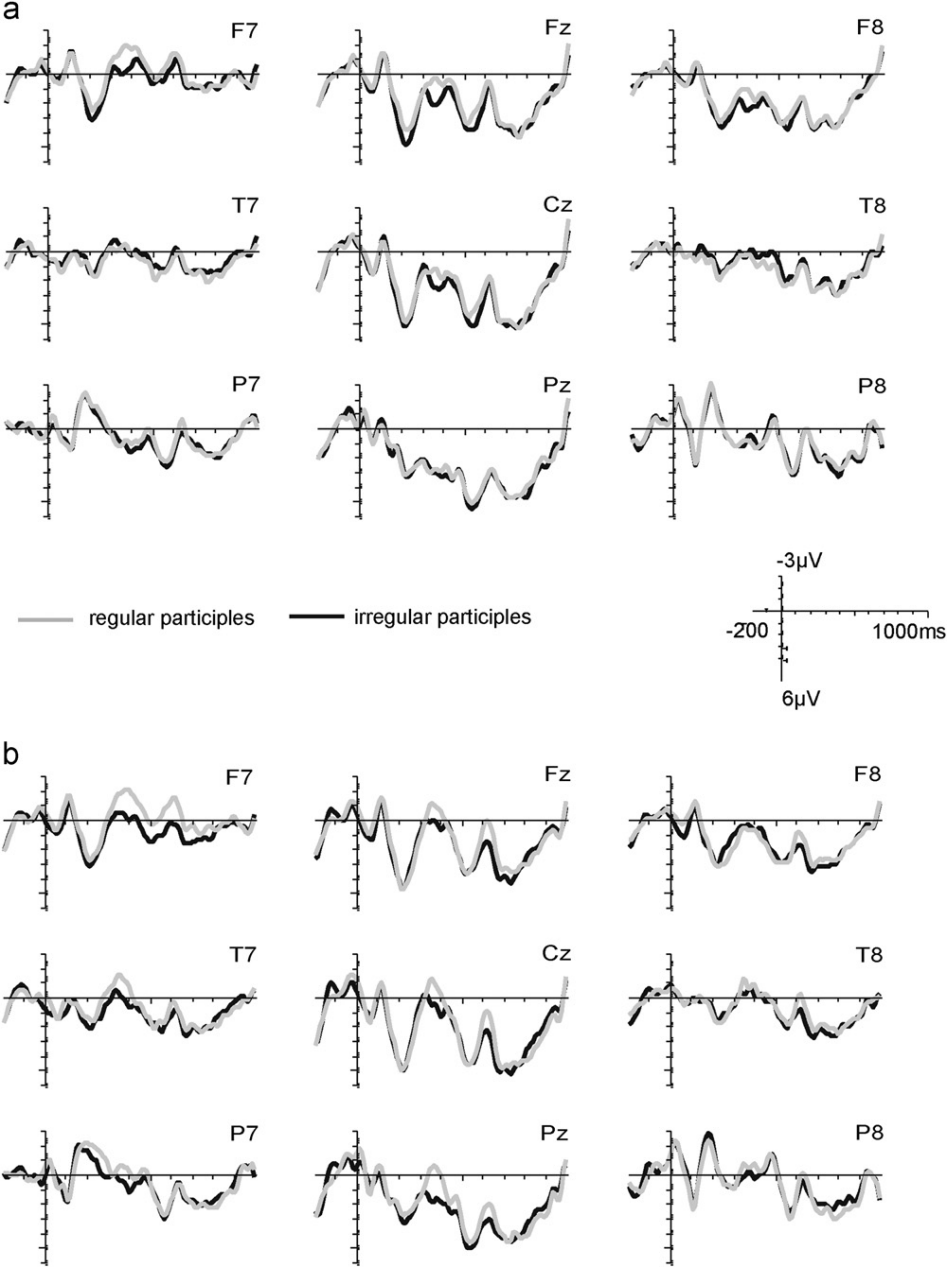
Grand average ERP waveforms for correct regular (grey) and irregular (black) participles for a subset of 9 electrodes representing the 9 ROIs (nose upward). (a) High frequency regular (RH) and irregular (IH) participles; (b) low frequency regular (RL) and irregular (IL) participles. For both frequency types, regular participles are associated with a larger negative-going (upward) deflection peaking at around 400 ms most pronounced over left anterior electrodes, i.e., a LAN.

**Fig. 3:**
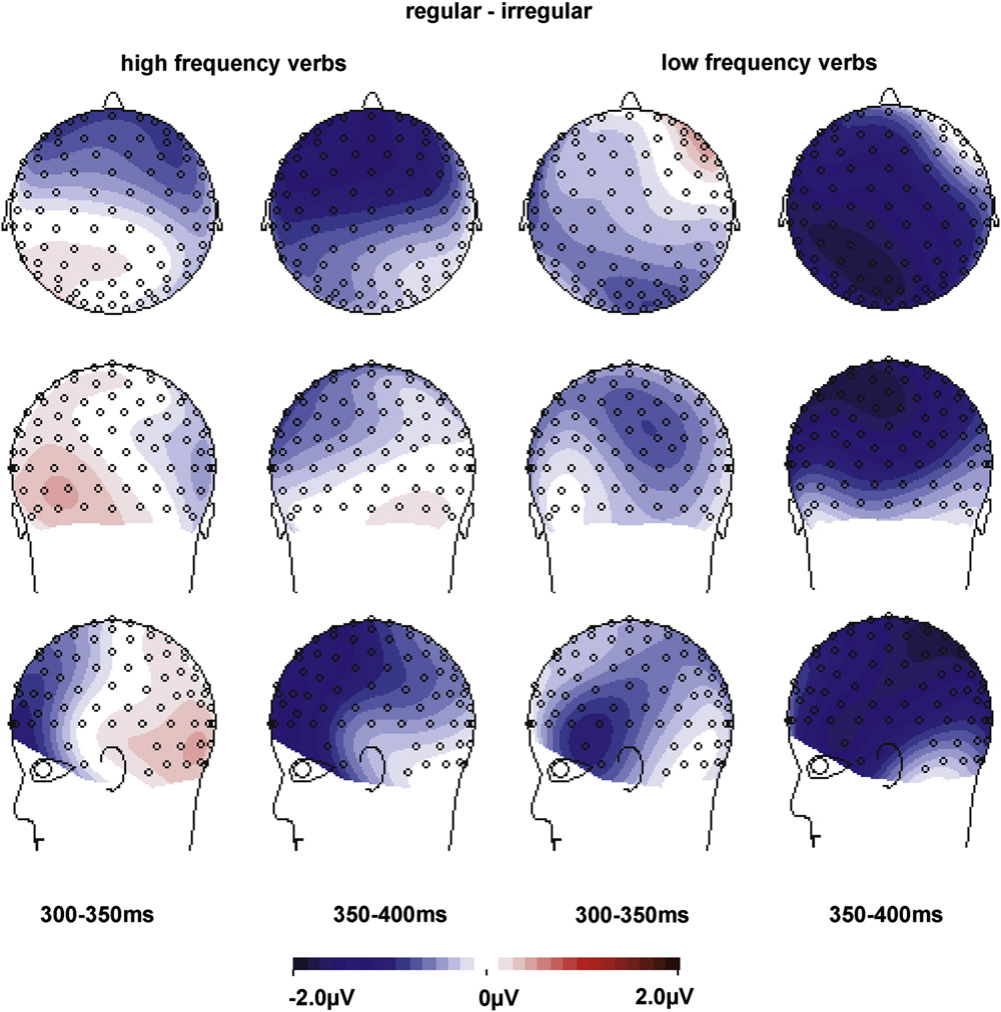
Spline-interpolated isovoltage maps for the regularity effects for high (left) and low (right) frequency participles. Maps are shown for the 300–400 ms time-window (left of vertical line) as well as for smaller 50 ms windows within the 300–400 ms time-window (right of vertical line). Maps show an increased left-anterior negativity for both high and low frequency regular participles compared to irregular participles. The effect for low frequency verbs stretches into posterior areas due to a co-occurring posterior-central source, visible in the 300–350 ms time-window.

The key questions of our study were whether correct regular verbs elicit LANs compared to correct irregular verbs and whether this regularity effect is modulated by frequency. We expected a LAN for the regularity contrast for low frequency verbs and, if rules are activated for high frequency verbs as well, also for high frequency verbs. Effects of the regularity manipulation are visible in the grand-average ERPs in Fig. 2a (high frequency verbs) and Fig. 2b (low frequency verbs). The topographic plots in Fig. 3 show the development of anterior left effects of regularity over the two time-windows, split by high and low frequency verbs. ANOVAs for each time-window (Table 2) revealed three-way interactions of Regularity×Frequency×ROI, suggesting differences in scalp distribution for the regularity effect for high and low frequency verbs. Fig. 3 suggests the emergence of a left anterior negativity for both high and low frequency verbs in the 300–350 ms time-window, with an additional posterior source for low frequency verbs. This was confirmed by analyses of variance with the factors Regularity and ROI for each Frequency level (Table 3), with a significant interaction of Regularity and ROI for the high frequency verbs. High frequency verbs showed trends for regularity difference at anterior ROIs (*α*=0.05/9=0.006; ROI1: *t*_(19)_=2.9, *p*=0.010; ROI2: *t*_(19)_=2.6, *p*=0.019; ROI3: *t*_(19)_=2.4, *p*=0.027) and low frequency verbs showed a trend at the left temporal ROI4 (*t*_(19)_=2.2, *p*=0.044). In the 350–400 ms time-window we found significant main effects of Regularity for both high and low frequency participles as well as interactions of Regularity with ROI (marginally significant for high frequency verbs). Follow-up analyses for each ROI (*α*=0.05/9=0.006) showed significant differences between regular and irregular verbs in left anterior ROIs for both high Frequency verbs (ROI1: *t*_(19)_=4.1, *p*=0.001; ROI4: *t*_(19)_=3.7, *p*=0.002; ROI5 *t*_(19)_=3.8, *p*=0.001) and low Frequency verbs (ROI1: *t*_(19)_=3.1, *p*=0.005; ROI4: *t*_(19)_=4.6, *p*<0.001; ROI5: *t*_(19)_=3.1, *p*=0.006), with additional effects at posterior left/central ROIs for low frequency verbs (ROI7: *t*_(19)_=3.1, *p*=0.006; ROI8: *t*_(19)_=3.4, *p*=0.003). Table 4 summarizes the effects for the two frequency levels in the left anterior ROI1 and the central posterior ROI8.

**Fig. 4:**
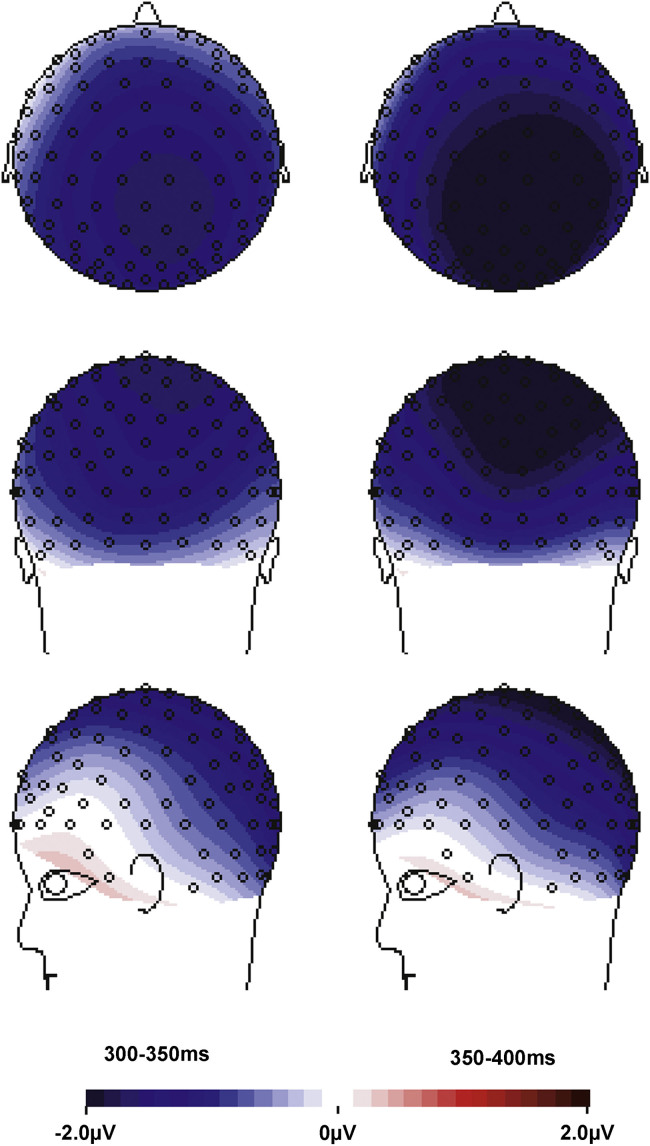
Spline-interpolated isovoltage maps for the main effect of frequency for the 300–400 ms time-window. Maps show the development of a typical frequency-related N400 effect with a posterior-right centre.

As expected for word frequency contrasts (e.g., Dambacher et al., 2006; Rugg, 1990; Van Petten and Kutas, 1990), our frequency manipulation led to a main effect of Frequency and an interaction of Frequency with ROI in both time-windows (Table 2), reflecting a more pronounced negative-going activity for low frequency than high frequency forms with a posterior-right scalp distribution, i.e., an N400. As visible in Fig. 4, the effect spread over all ROIs, except for the left anterior ROIs 1, 2, and 4 (e.g., central ROI5: 300–350 ms: high frequency: 1.2 μV, SD 1.9, low frequency: −.4 μV, SD 2.8; 350–400 ms: high frequency: 1.8 μV, SD 1.4, low frequency: −.1 μV, SD 2.4). Most importantly, the presence of an N400 confirmed that the frequency contrast in our stimuli was large enough to enable the detection of different neural processing with regards to regularity.

### 2.2 Incorrect vs. correct participle forms

**Fig. 5:**
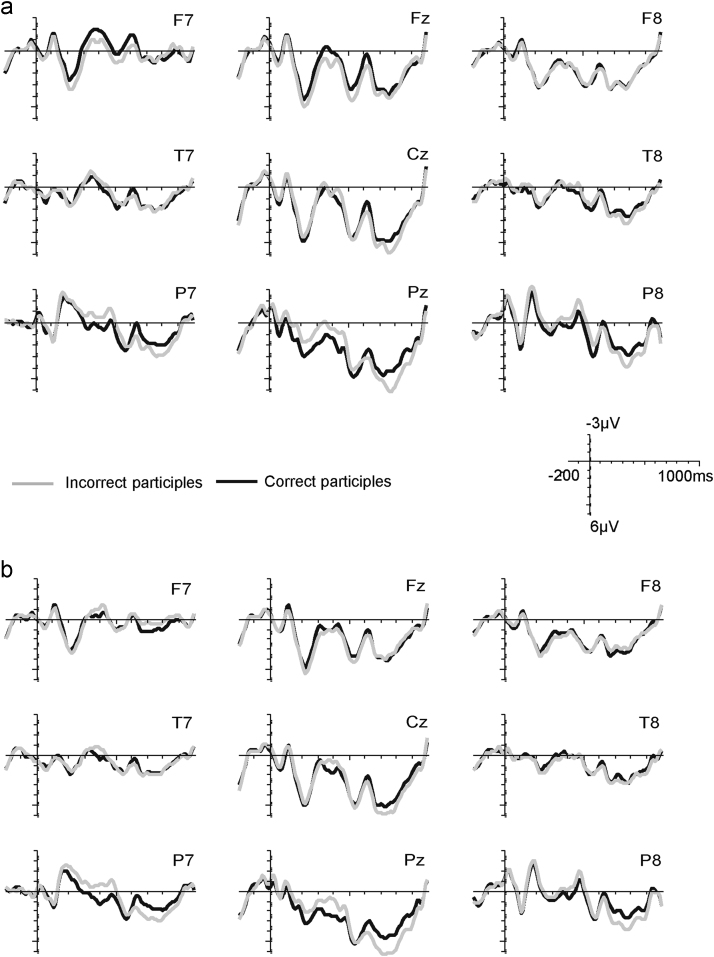
Grand average ERP waveforms for correct (black) and incorrect (grey) participles for a subset of 9 electrodes representing the 9 ROIs (nose upward). (a) Regular participles, (b) irregular participles. For both participle types, incorrect participles are associated with a larger negative-going (upward) deflection at posterior sites. This negativity spreads into left temporal sites for irregular participles. Waveforms also show late posterior positivities for both incorrect regular and irregular verbs, which are not in the focus of this study and are therefore not analysed.

**Fig. 6:**
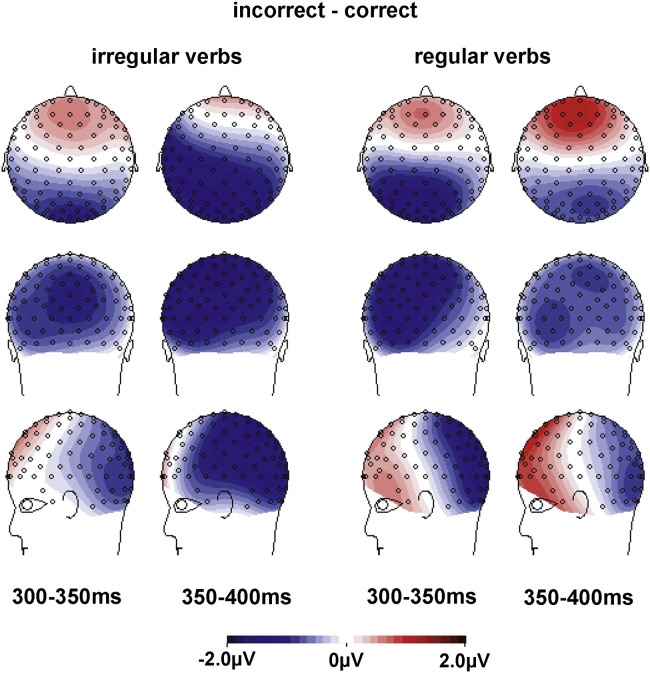
Spline-interpolated isovoltage maps for the Correctness effects for irregular (left) and regular participles (right). Maps show posterior negativities (N400s) for both verb types. In addition, incorrect irregular participles show a co-occurring left temporal negativity (a variation of the LAN) in the 350–400 ms time-window.

To determine whether our data showed a LAN for overregularisation of irregular past participles, as reported by Penke et al. (1997), we conducted ANOVAs with the factors Regularity, Correctness, and ROI for both time-windows.^2^ Table 5 shows an interaction of Regularity×Correctness×ROI for both windows, suggesting that the use of incorrect suffixes led to different brain responses for regular compared to irregular verbs. This is illustrated in Figs. 5 and 6. While incorrect forms led to negativities for both regular and irregular verbs, the distribution and the robustness of these negativities differed. For irregular verbs, the effect spread over left temporal and posterior electrodes. This effect appeared to be a combination of (at least) two components, a central posterior one that is visible in both time-windows, and a left temporal one that spreads into some left anterior electrodes and that is only present in the 350–400 ms time-window. In contrast, the negativity for regular participles is confined to posterior electrodes. These topographic differences are reflected in separate ANOVAs for regular and irregular participles (see Table 6). For both verb types, we found significant interactions of Correctness with ROI in both time-windows. In case of irregular verbs, comparisons of correct and incorrect forms revealed trends for differences at posterior ROIs (*α*=0.05/9=0.006; ROI7: *t*_(19)_=2.1, *p*=0.045; ROI8: *t*_(19)_=2.2, *p*=0.039) in the 300–350 ms time-window. In the 350–400 ms time-window, significant differences were found at posterior ROIs (*α*=0.05/9=0.006; ROI7: *t*_(19)_=5.7, *p*<0.001; ROI8: *t*_(19)_=5.4, *p*<0.001; ROI9: *t*_(19)_=3.4, *p*=0.003) as well as at the left temporal ROI4 (*t*_(19)_=4.4, *p*<0.001). For regular verbs, comparisons of correct and incorrect regular forms showed trends for differences at posterior sites in both time-windows (*α*=0.05/9=0.006; 300–350 ms: ROI8: *t*_(19)_=2.5, *p*=0.023, ROI9: *t*_(19)_=2.1, *p*=0.054; 350–400 ms: ROI8: *t*_(19)_=2.0, *p*=0.055). Table 7 summarizes these effects in the left temporal ROI4 and the central posterior ROI8.

## 3 Discussion

This study addressed the question whether LANs reflect merely processing the violation of rules or rule application per se by investigating whether LANs occur in correctly formed verbs. In addition, we asked whether a LAN as evidence for rule application can only be found for low-frequency regular forms, as predicted by earlier behavioral studies. To answer these questions we presented correct and incorrect high and low frequency German past participles with varying regularity, while simultaneously recording EEG.

Electrophysiological responses to correct regular and irregular German participles revealed distinct patterns: compared to correct irregular participles, correct regular participles elicited more negative activity over the left tempo-frontal scalp area (LAN) at around 350–400 ms after stimulus onset. This left anterior negativity was present for both high and low frequency verbs. Because (a) regular and irregular items were matched on all other factors but regularity (i.e., full-form frequency, lemma frequency, length, etc.), and (b) LANs have been found for misapplications of rules in this and previous studies, we conclude that our LAN for correct regular verbs reflects rule application, which is present for regular participles but not for irregular participles. Because we found a LAN for correct forms, it cannot be attributed to error handling.

Correct low frequency items showed a negative activation pattern that reached into posterior-left areas. This appeared to be due to two co-occurring activities from an anterior and a posterior source, visible in the activation pattern in the 300–350 ms time-window and supported by the finding that differences between low frequency regular and irregular participles were significant in left temporal and central posterior electrode clusters in the 350–400 ms window. The additional posterior activity is difficult to interpret and requires further research. But the mere fact that that there is a difference between low frequency regular and irregular participles supports the hypothesis that those are not processed in exactly the same way.

A comparison of high and low frequency participles showed a classical N400 effect (e.g., Dambacher et al., 2006; Rugg, 1990; Van Petten and Kutas, 1990). This confirms that our frequency contrast was large enough to detect processing differences for high and low frequency participles with regards to regularity. In other words, we can be confident that our frequency manipulation was successful and LANs were indeed present independent of the frequency status of the verbs.

Effects for incorrect compared to correct participles were somewhat different from what had been found by Penke et al. (1997), but are still in line with their conclusions. Incorrect irregular participles elicited not only a frontotemporal negativity, but two co-occurring negative components, a left temporal and a central posterior one. The left-temporal component was present during the same time-window as the LAN for correct regular participles and is therefore likely a variation of the same component reflecting rule processing, even though it is not as anterior. The co-occurring posterior central negativity might have altered the location of the LAN to more temporal sites. Note also that a left temporal negativity has been found for the misapplication of stem formation rules in Catalan (Rodriguez-Fornells et al., 2001), which means that the location of the LAN for incorrect rule application in our study is not that unusual.

In contrast to Penke et al. (1997) study, we found a posterior negativity for both incorrect irregular and regular participles, even though it was not quite significant for regular verbs. Since it occurred for forms that are not part of the language, it is most likely an N400, known to occur for nonwords and novel complex words (e.g., Deacon et al., 2004; Krott et al., 2006; Lehtonen et al., 2007; Leinonen et al., 2009). The fact that N400s are only found in our study, but not in that by Penke et al. (1997) might be due to the different tasks. While participants were asked to judge the correctness of sentences in our study, Penke et al. (1997) used memory tasks and a noun detection task. Participants might have been more sensitive to the non-existence of the incorrect participles when asked to judge their correctness in our study.

The key findings of our study are that (a) regular inflected forms are processed differently from irregular ones, indicated by a LAN, and that (b) a LAN can be elicited by correct forms. Our findings therefore validate the claim in the previous literature that the processing of regular forms operates via a distinct neural substrate. Crucially, the current results imply that LANs found when rule applications were violated (Gross et al., 1998; Linares et al., 2006; Penke et al., 1997; Rodriguez-Fornells et al., 2001; Weyerts et al., 1997) indeed reflected the application of rules rather than merely processes related to the violation and/or the mismatch of a present incorrect form of a word with its stored correct form (Krott et al., 2006). Thus, our findings, along with previous findings on LANs, support dual-mechanism approaches, which assume a rule application mechanism that is activated for regular forms (e.g., Clahsen, 1999; Pinker, 1999a; Pinker and Ullman, 2002; Prasada and Pinker, 1993; Ullman, 2001).

However, the finding that high frequency participles showed a LAN does not completely fit with traditional dual-mechanism models. It has been argued that high frequency regular inflections are stored and accessed via full-form representations just like irregular inflections (e.g., Alegre and Gordon, 1999; Baayen et al., 1997; Bertram et al., 2000; New et al., 2004; Schreuder and Baayen, 1995; Sereno and Jongman, 1997). And dual-mechanism accounts have assumed that stored forms block the application of a rule (e.g., Clahsen et al., 2004; Marcus et al., 1995; Pinker, 1999b). Therefore, high frequency regular participles should have blocked the rule and should not have shown a LAN. The fact that they did suggests that regular forms activate rule processing mechanisms independently of whether they have full-form representations or not, contrary to what has been argued previously (e.g., Clahsen et al., 2004; Marcus et al., 1995; Pinker, 1999b). What might be happening is that the two routes of a dual-mechanism model, the full-form access route and the decomposition route, are activated in parallel for high frequency forms. This parallel activation may not have been detected by response time measures in previous behavioral studies because the faster full-form route might have won the race of lexical access and determined response times (e.g., Alegre and Gordon, 1999; Baayen et al., 1997; Bertram et al., 2000; New et al., 2004; Schreuder and Baayen, 1995; Sereno and Jongman, 1997).

The current results also address the question as to whether LANs reflect rule application or decomposition of a complex word into its constituents. In languages like English it is difficult to tease apart rule application and decomposition because decomposability and regularity go hand in hand (see irregular *went, saw, was* in contrast to regular *walk+ed, watch+ed, appear+ed*). However, in German, it is possible to observe the effect of rule application per se because regular and irregular participles are both decomposed (Smolka et al., 2007). Thus, our results indicate that LANs do reflect rule application, not decomposition.

Because both regular and irregular German participles are decomposed (Smolka et al., 2007), we do not propose a model, in which regular and irregular inflected forms are processed by either decomposition or full-form access and that one route excludes the other. Instead, we argue for a parallel route model, in which an additional rule mechanism is activated by regular forms, but that this route does not necessarily determine the speed of lexical access.

Our findings pose a challenge to single-mechanism theories that assume that the processing of regular formations is not subserved by a distinct neural substrate (e.g., Joanisse and Seidenberg, 2005; McClelland and Patterson, 2002; Rumelhart and McClelland, 1986; Smolka et al., 2007). Proposers of single-mechanism models could argue that the LAN in our study might have been caused by other features of the participles rather than their regularity status, e.g., phonological differences or differences in type frequency of *-*(*e*)*t* and *-*(*e*)*n* (for discussions of type frequency see Clahsen, 1999; Marcus et al., 1995; Smolka et al., 2007). However, systematic phonological differences do not seem to exist (e.g., Beedham, 1994) and LANs have been found for both minority suffixes (in terms of type frequency) such as the German plural suffix *-s* (Weyerts et al., 1997) and majority suffixes such as the English past tense suffix -*ed* (Morris and Holcomb, 2005), which means that differences in type frequency cannot explain the occurrence of LANs.

In summary, we have provided evidence that LANs reflect processes involved in rule application because they are elicited by correctly formed regular participles. This supports the assumption that regular and irregular participles are handled by at least partially distinct neural substrates during word processing. Our results are therefore in accordance with dual-mechanism models of morphological processing that assume a separate mechanism for regular inflection. We also found that LANs are elicited by regular participles regardless of their frequency. Thus, in contrast to traditional dual-mechanism models, we argue for a parallel route model, in which a separate rule mechanism is activated independently of full-form storage and in which inflected words can be processed by more than one route simultaneously.

## 4 Experimental procedure

### 4.1 Pre-test

#### 4.1.1 Methods

##### 4.1.1.1 Materials

The selection of German past participles to be used as experimental material was based on a behavioral pre-test that (a) determined that participants are familiar with the correct forms of both regular and irregular participles and (b) investigated whether these participles were processed with equal ease. The material and procedure of the pre-test were kept as close as possible to those of the EEG-experiment. Because the time-window analyzed in the EEG experiment (see below) did not allow any conscious reflection on the participles, we used a speeded grammatical judgment task for the pre-test.

Participants judged the correctness of 138 regular (69 high-frequency and 69 low-frequency) and 122 irregular (61 high-frequency and 61 low-frequency) German past participles. As mentioned, regular and irregular participles differed only in terms of the suffix, i.e., *-*(*e*)*t* for regular forms and *-*(*e*)*n* for irregular forms. All contained the prefix *ge-* and in all forms the stem vowel of the present tense was preserved (e.g., regular *ge-glaub-t* and irregular *ge-fahr-en*). Due to a limited number of such verbs, we included verbs with stems that consisted of a simple root (e.g., *glaub*) or of a root plus prefix (e.g., *los+lauf*). We presented both correct participles and their corresponding incorrect forms. For the latter we replaced the regular *-*(*e*)*t* suffix with the irregular *-*(*e*)*n* suffix and vice versa (e.g., correct regular *geglaubt* ‘believed’>incorrect *geglauben*, correct irregular *gefahren* ‘traveled’>incorrect *gefahrt*).

Participles were presented at the final position of a 7-word sentence. An example of a correct and incorrect sentence is given in (1), where ‘⁎’ marks the incorrect form. Finally, we created 14 practice sentences containing 7 correct and 7 incorrect past participles that were not used in the experiment.

(1) a. Die Eltern sind in den Schwarzwald gefahren.
  ‘The parents traveled to the Black Forest’.
b. Die Eltern sind in den Schwarzwald ^⁎^gefahrt.
  ‘The parents travel+IncorrectSuffix to the Black Forest’.

##### 4.1.1.2 Participants

Twenty-two monolingual native German speakers, recruited at Bielefeld University and with normal or corrected-to-normal vision, were paid for their participation in the pre-test (13 females, mean age=24.7 years, SD=2.0).

##### 4.1.1.3 Procedure

The experimental sentences were presented word by word in black letters against a white background using the E-Prime software. Each trial began with a fixation cross appearing in the centre of the screen for 800 ms, followed by a 200 ms blank screen. Subsequently, each word was presented in the centre for 300 ms, followed by 150 ms blank screen. Participants were instructed to decide as quickly and as accurately as possible whether the participle was correct or not by pressing the ‘Yes’ or ‘No’ button of a button box. ‘Yes’ responses were always given by the dominant hand. Target words, i.e., participles, were presented in italics to initiate responses. Maximum response time was 2000 ms, measured from the onset of the target. The following trial was initiated by the response.

All participants saw all experimental sentences, which were divided into 5 blocks. The order of the blocks was counterbalanced across participants and the proportion of stimulus types was equal in all blocks. Each participant saw a different randomized order of the items within each block. Correct and incorrect forms of a given sentence were never both present within a single block. Moreover, participle variations (i.e., correct and incorrect) were presented with an interval of at least two experimental blocks. Prior to the experiment, participants saw the practice sentences to familiarize themselves with the task. After each experimental block (including the practice block), feedback on the computer screen reported mean accuracy and mean reaction time. Participants were asked to adapt their behavior according to the feedback, i.e., to speed up when responding too slowly or to be more accurate when making a lot of mistakes.

#### 4.1.2 Results

Error rate and reaction time to correct participles were submitted to repeated measures analyses of variance (ANOVA; *F*_1_: by-subject analysis; *F*_2_: by-item analysis^3^) with the factors Regularity (regular vs. irregular) and Frequency (high frequency vs. low frequency).

An analysis of error rates (ERs) revealed a main effect of Frequency (*F*_1(1,21)_=16.0, *p*=0.001; *F*_2(1,256)_=21.0, *p*<0.001), with significantly more mistakes for low frequency participles (mean ER=3.4%, SD=3.3) than for high frequency ones (mean ER=1.3%, SD=1.7). Neither a main effect of Regularity was found (regular participles: mean ER=2.6%, SD=3; irregular participles: mean ER=2.2%, SD=2.7), nor was there any significant interaction (*p*>0.05). While these results showed that participants made slightly more errors to low frequency participles than to high frequency ones, the overall ER was very low. This means that the correct forms of the participles used in our experiment were very well known.

The analysis of reaction times (RTs) mirrored those of error rates, revealing a main effect of Frequency (*F*_1(1,21)_=331.0, *p*<0.001; *F*_2(1,256)_=88.0, *p*<0.001), with participants being significantly faster to respond to high frequency participles (mean RT=739.8 ms, SD=74.9) than to low frequency ones (mean RT=827 ms, SD=76.5). Neither a main effect of Regularity was found (regular participles: mean RT=783.3 ms, SD=88.9; irregular participles: mean RT=783.6, SD=86.3), nor was there any significant interaction (*p*>0.05). RTs were in accordance with the typical finding that high-frequency words are recognized faster than low-frequency words. Error rates and reaction times together showed that regular and irregular participles were processed with equal efficiency.

### 4.2 EEG experiment

#### 4.2.1 Methods

##### 4.2.1.1 Materials

Out of the pre-tested participles we selected 100 regular and 100 irregular German past participles, i.e., four participle categories with 50 items each: high frequency irregular participles (IH), low frequency irregular participles (IL), high frequency regular participles (RH), and low frequency regular participles (RL) (see Appendix A).

As shown in Table 8, participles were matched for length, lemma frequency and full-form frequency on both frequency levels, with equal error rates and reaction times (independent sample *t*-tests, two-tailed, all *p*>0.05). High and low frequency participles did not differ in terms of length (*p*>0.05), but in terms of both full-form frequency (*t*_(98)_=24.4, *p*<0.001) and lemma frequency (*t*_(98)_=17.0, *p*<0.001). The latter was necessary to select a sufficient number of experimental items. Frequency measures are taken from the CELEX lexical database (Baayen et al., 1995). We logarithmically transformed frequency measures due to their skewed distributions.

We presented the participles embedded in the same sentences as in the pre-test, followed by a sub-ordinate clause as shown in (2). The sub-ordinate clause prevented an overlap of ERP effects on the participle with sentence-final wrap-up effects (Hagoort et al., 2003; Osterhout and Holcomb, 1992). As in the pre-test, incorrect forms of the participles were used as fillers.

(2) a. Die Eltern sind in den Schwarzwald gefahren, um Oma zu sehen.
  ‘The parents travelled to the Black Forest to see grandma.’
b. Die Eltern sind in den Schwarzwald ^⁎^gefahrt, um Oma zu sehen.
  ‘The parents travel+IncorrectSuffix to the Black Forest to see grandma.’

##### 4.2.1.2 Participants

Twenty native German speakers participated in the EEG experiment (12 females, mean age=22.9 years, SD=1.4). All were paid and gave their informed consent. All participants were right-handed as assessed by a German version of the Edinburgh handedness inventory (Oldfield, 1971). No subject had a history of neurological/psychiatric disorders or reported reading difficulties. All had normal or corrected-to-normal vision.

##### 4.2.1.3 Procedure

The procedure of the EEG experiment was very similar to that of the pre-test. Participants were seated in front of a 17″ monitor with a resolution of 800×600 pixels with a viewing distance of 1 m. Each trial began with a fixation cross appearing in the centre of the screen for a varying period of time, randomly ranging from 800 to 1100 ms, and followed by a 100 ms blank screen. As in the behavioral experiment, words were then presented one by one in the middle of the screen for 300 ms, followed by 150 ms blank screen. The visual angle varied from 1.7 to 4.0°. The participle was presented in normal font, not in italics as in the pre-test. Commas separating main clauses from sub-ordinate clauses were treated as words, i.e., they were presented independently for 300 ms with following 150 ms blank screen. To reduce the length of the experiment, subordinate clauses were presented as wholes for 1000 ms in the centre of the screen. Following the offset of a sentence, a 2400 ms blank screen allowed participants to blink or move. Trials lasted on average for 8500 ms. The procedure for presentation order and feedback was the same as in the pre-test.

A correctness judgment task was used to ensure that participants attended to the stimuli. For 1/3 of the trials, the final blank screen of a trial was reduced to 400 ms, after which a question mark appeared in the centre of the screen. The question mark was visible for a maximum duration of 2000 ms. At this point, the participants’ task was to decide whether the preceding sentence was correct or not, indicating their judgment with a button-press on a response box. The hand used to give the ‘Yes’ response was counterbalanced across participants. The items responded to were randomly selected, yet equally distributed over target stimuli and fillers. Moreover, we ensured that the probability of a response word being a word vs. a nonword was 50:50. The question mark disappeared with the response, leaving the screen blank for the remaining time of the trial.

##### 4.2.1.4 EEG recording parameters

EEG was recorded continuously from 128 Ag/AgCl scalp electrodes relative to an (off-line) averaged left and right mastoid reference. The electrodes were placed according to the extended 10–20 electrode system (Oostenveld and Praamstra, 2001) using a nylon electrode cap. Eye movements were monitored by bipolar horizontal and vertical EOG derivations. EEG and electro-oculography (EOG) signals were amplified with a band-pass of 0.16–100 Hz by BioSemi Active-Two amplifiers and sampled at 512 Hz. The continuous EEG recordings were segmented off-line in 1200 ms epochs starting 200 ms prior to past participle onsets and spanning 1000 ms afterwards. Epochs containing artifacts from amplifier blocking, signal drifts, excessive eye movements (including blinks), or muscle activity were rejected off-line before averaging. On average, 4.6% (±3.9) of trials were lost due to such artifacts. Averages of artifact-free event-related potentials (ERPs) were calculated for each type of target category (e.g., correct regular and irregular participles) after subtraction of the 200 ms pre-stimulus baseline. Prior to measurement, ERPs were digitally filtered with a band-pass of 0.2–20 Hz.

## Figures and Tables

**Table A1:** Past participles used as stimuli in the EEG experiment.

Regular participles, high frequency	Regular participles, low frequency	Irregular participles, high frequency	Irregular participles, low frequency
geöffnet	festgenäht	gelesen	gehauen
geliefert	aufgelockert	gelassen	zugehalten
gesetzt	abgespült	eingesehen	angelesen
gestürzt	gestülpt	getragen	hingesehen
ausgebildet	angeheuert	gefangen	ausgeblasen
aufgeführt	umgeklappt	weitergegeben	losgefahren
gerichtet	eingereist	geschaffen	umgeladen
dargestellt	zugesteuert	beigetragen	nachgelesen
gekauft	abgeklopft	eingehalten	abgefressen
geschildert	geschmaust	geschlagen	aufgelaufen
ausgeliefert	gelockert	geladen	rangehalten
kennengelernt	gekrümelt	vorgekommen	gegraben
ausgebaut	kleingehackt	angefangen	aufgeblasen
eingestellt	gestriegelt	eingetragen	abgemessen
gebildet	gejodelt	aufgegeben	geblasen
ausgezeichnet	nachgezahlt	gewachsen	weggetreten
gelernt	eingelebt	eingeladen	umgegraben
gewechselt	gewürfelt	getreten	hingetragen
gerettet	zugeprostet	gerufen	losgekommen
geprüft	angenäht	gemessen	umgehauen
geglaubt	abgeklemmt	aufgehalten	weggefallen
geklärt	umgeknickt	festgehalten	achtgegeben
durchgeführt	zugeklebt	angehalten	umgefallen
geschickt	gesattelt	eingeschlagen	dagelassen
geeignet	gejubelt	freigegeben	angebraten
eingerichtet	geräuchert	hervorgerufen	ausgeladen
geschützt	geschnallt	vorgeschlagen	eisgelaufen
gerechnet	zugenäht	geraten	ausgekommen
gespielt	ausgespült	gesehen	ausgelesen
gesteigert	gespurtet	ausgetragen	eingelesen
eingeschätzt	gerostet	eingeschlafen	vollgeladen
gebraucht	untergejubelt	ausgegeben	umgeblasen
geschenkt	umgesattelt	geschlafen	vorgeladen
gezeigt	abgezahlt	gewaschen	abgewaschen
geantwortet	gedrosselt	aufgefallen	hergebeten
gewartet	eingewickelt	eingetreten	weggestoßen
getrennt	abgewickelt	aufgetreten	rotgesehen
gestützt	aufgetakelt	vorgetragen	klargesehen
geäußert	gebüffelt	gefahren	gebacken
gemeint	gemeckert	abgelaufen	angehauen
gefördert	zugeklappt	herausgegeben	gebraten
durchgesetzt	geschwelgt	abgesehen	losgelaufen
eingeleitet	angekohlt	gekommen	aufgegraben
geleistet	geködert	angekommen	abgegraben
fortgesetzt	geschwemmt	angesehen	zugelaufen
gemeldet	umgemodelt	gelaufen	aufgehauen
ausgeführt	aufgeklappt	zugegeben	hingefallen
gekümmert	abgeknickt	gehalten	hergegeben
ausgelöst	angemahnt	zugelassen	abgehauen
gewidmet	angezahlt	zurückgetreten	vorgetreten

**Table 8:** Properties of regular and irregular participles used in the EEG experiment.

Property	Regularity	Frequency			
		High		Low	
		Mean	SD	Mean	SD

Length in letters	Irregular	10.0	1.9	10.1	1.1
	Regular	9.6	1.7	10.0	1.1
Log full-form frequency	Irregular	1.99	0.37	0.29	0.33
	Regular	2.09	0.25	0.21	0.22
Log lemma frequency	Irregular	2.55	0.55	0.7	0.6
	Regular	2.66	0.38	0.5	0.4
Error rates	Irregular	0.8	1.7	2.7	2.8
	Regular	1.0	1.2	2.5	3.7
Reaction times	Irregular	736.2	77.8	814.5	77.6
	Regular	736.4	73.2	820.0	74.9

**Table 1:** Regions of interest (ROIs) and their corresponding pooled electrodes from a 128-channel layout arranged along the extended 10–20 system (Oostenveld and Praamstra, 2001).

Region Of interest (ROI)	Pooled electrodes	ROI position
ROI_1_	AF7, AF5h, AFF7h, AFF5h, F7, F5, F3, FFT7h, FFC5h, FFC3h	Left anterior site
ROI_2_	FP1, FPz, FP2, AF3h, AFz, AF4h, F1, Fz, F2, FFC1h, FFC2h	Centro-anterior site
ROI_3_	AF8, AF6h, AFF8h, AFF6h, F8, F6, F4, FFT8h, FFC6h, FFC4h	Right anterior site
ROI_4_	FT7, FC5, FC3, FTT7h, FCC5h, T7, C5, C3, TTP7h, CCP5h, TP7, CP5, CP3	Left central site
ROI_5_	FC1, FCz, FC2, FCC3h, FCC1h, FCC2h, FCC4h, C1, Cz, C2, CCP3h, CCP1h, CCP2h, CCP4h, CP1, CPz, CP2	Central site (vertex)
ROI_6_	FT8, FC6, FC4, FTT8h, FCC6h, T8, C6, C4, TTP8h, CCP6h, TP8, CP6, CP4	Right central site
ROI_7_	TPP7h, CPP5h, CPP3h, P7, P5, P3, PPO5h, PO5h, PO7	Left posterior site
ROI_8_	CPP1h, CPP2h, P1, Pz, P2, PPO3h, PPO1h, PPO2h, PPO4h, PO3h, POz, PO4h, POO1, POO2, O1, Oz, O2	Centro-posterior site
ROI_9_	TPP8h, CPP6h, CPP4h, P8, P6, P4, PPO6h, PO6h, PO8	Right posterior site

**Table 2:** Statistical results for correct participles for the two time-windows. Values: statistical index *F* and its corresponding *p* value; factors: regularity (Reg: irregular, regular), frequency (Freq: high frequency, low frequency), ROI (9 regions: cf. Table 1); “AxB”=interaction of factors A and B.

Time window	Reg	Freq	Reg x Freq	Reg xROI	Freq xROI	Reg x Freq xROI
300–350 ms						
* F*	3.4	19.5	.0	1.6	5.4	4.0
* p*	.081	<.001	.858	.125	.003	.028
350–400 ms						
* F*	14.6	25.6	1.1	3.3	4.2	5.1
* p*	.001	<.001	.3.08	.036	.015	.014

**Table 3:** Statistical results for the regularity and ROI factors for high and low frequency correct participles in the two time-windows. Values: statistical index *F* and its corresponding *p* value; factors: regularity (Reg: irregular, regular), ROI (9 regions: cf. Table 1); “A×B”=interaction of factors A and B.

Time window	High frequency participles		Low frequency participles	
	Reg	Reg×ROI	Reg	Reg×ROI

300–350 ms				
* F*	2.3	4.1	1.6	1.9
* p*	.143	.014	.216	.160
350–400 ms				
* F*	9.1	3.0	10.2	6.0
* p*	.007	.062	.005	.002

**Table 4:** Mean μV and standard deviations (in parentheses) for the anterior left electrode cluster ROI1 and the posterior central electrode cluster ROI8 for correct regular (reg) and correct irregular (irr) participles, split into high and low frequency verbs and the two time-windows. Significant differences between regular and irregular participles are indicated in bold, while trends are indicated in italics.

	300–350 ms				350–400 ms			
	ROI1		ROI8		ROI1		ROI8	
	reg	irr	reg	irr	reg	irr	reg	irr
High frequency	−*.6* (*1.9*)	*.4* (*1.9*)	2.2 (1.8)	−.1 (1.6)	−**1.0** (**1.9**)	**.6** (**2.2**)	2.4 (2.1)	2.6 (2.5)
Low frequency	−*.7* (*2.3*)	*2.1* (*2.0*)	.6 (2.4)	1.3 (2.3)	−**1.4** (**2.4**)	−**.1** (**1.8)**	**0** (**1.9)**	**1.7** (**2.7)**

**Table 5:** Statistical results for all participle forms for the two time-windows. Values: statistical index *F* and its corresponding *p* value; factors: regularity (Reg: irregular, regular), correctness (Corr: correct, incorrect), ROI (9 regions: cf. Table 1); “A×B”=interaction of factors A and B.

Time window	Reg	Corr	Reg×Corr	Reg×ROI	Corr×ROI	Reg×Corr×ROI
300–350 ms						
* F*	3.4	1.7	.9	6.6	5.6	3.5
* p*	.080	.208	.346	.002	.002	.025
350–400 ms						
* F*	5.2	1.9	.01	3.5	4.7	3.3
* p*	.035	.183	.905	.041	.011	.039

**Table 6:** Statistical results for the Correctness factor for regular and irregular participles in the two time-windows. Values: statistical index *F* and its corresponding *p* value; factors: correctness (Corr: correct, incorrect), ROI (9 regions: cf. Table 1); “A×B”=interaction of factors A and B.

Time window	Regular participles		Irregular participles	
	Corr	Corr×ROI	Corr	Corr×ROI
300–350 ms				
* F*	1.3	4.3	.1	5.7
* p*	.260	.010	.727	.005
300–350 ms				
* F*	.5	3.6	5.7	9.3
* p*	.488	.029	.027	<.001

**Table 7:** Mean μV and standard deviations (in parentheses) for the temporal left electrode cluster ROI4 and the posterior central electrode cluster ROI8 for correct (cor) and incorrect (incor) participles, split into regular and irregular verbs and the two time-windows. Significant differences between correct and incorrect participles are indicated in bold, while trends are indicated in italics.

	300–350 ms				350–400 ms			
	ROI4		ROI8		ROI4		ROI8	
	cor	incor	cor	incor	cor	incor	cor	incor
Regular	−.8 (3.5)	.6 (2.0)	*2.8* (*3.7)*	−*.3* (*2.2)*	−1.1 (2.9)	.5 (1.9)	*2.4* (*3.5)*	−*.4* (*2.7)*
Irregular	0 (1.5)	−.2 (1.8)	*1.7* (*2.0)*	*.9* (*1.9)*	**.6** (**1.5)**	−**.3** (**1.8)**	**2.2** (**2.5)**	**.8** (**2.6)**

## Appendix A

Please see Table A1.

## Footnotes

2 We also checked whether the differences between correct and incorrect participle forms were affected by the frequency of the correct form. But ANOVAs with the factors regularity, correctness, frequency and ROI did not show any interaction of frequency with regularity and correctness for any of the time-windows. This suggests that the frequency of the correct forms did not matter.

3 *F*_1_ refers to an *F*-test that treats participants as a random factor and items as fixed. It tests whether the results can be generalized to other subjects by averaging responses to all items in each condition for each participant. *F*_2_ refers to an *F*-test that treats items as a random factor and subjects as fixed. It tests whether the results can be generalized to other items by averaging responses from all participants for each item.
